# A review of elasmobranch catch-and-release science: synthesis of current knowledge, implications for best practice and future research directions

**DOI:** 10.1093/conphys/coad100

**Published:** 2023-12-28

**Authors:** Luke W J Cameron, William K Roche, Katy Beckett, Nicholas L Payne

**Affiliations:** School of Natural Sciences, Department of Zoology, Trinity College Dublin, Dublin 2, Ireland; Inland Fisheries Ireland, 3044 Lake Drive, Citywest Business Campus, Dublin D24 CK66, Ireland; School of Natural Sciences, Department of Zoology, Trinity College Dublin, Dublin 2, Ireland; School of Natural Sciences, Department of Zoology, Trinity College Dublin, Dublin 2, Ireland

**Keywords:** Batoidea, Carcharhiniformes, Chondricthyes, Lamniformes, Rajiformes, ray, recreational angling, rod and line, Selachimorpha, shark

## Abstract

Until relatively recently commercial fisheries have been considered the main driving factor for elasmobranch population declines. However, this belief has begun to shift with the realization that recreational elasmobranch catches may equal or exceed commercial catches in some regions. Many recreational angling fisheries for elasmobranchs involve high participation in catch-and-release angling practices. However, high release rates may not necessarily equate to high survival rates. Therefore, to assist accurate assessment of the potential impact of recreational angling on elasmobranchs, we attempted to summarize and integrate currently available information on specific risk factors associated with recreational angling, alongside associated mortality rates, as well as information on angler behaviour as it relates to identified risk factors. We categorized the major angling-related effects into two groups: injury-induced effects; and biochemical disruption-induced effects; providing a summary of each group and outlining the main lethal and sub-lethal outcomes stemming from these. These outcomes include immediate and delayed post-release mortality, behavioural recovery periods (which may in-turn confer increased predation risks), chronic health impacts and capture-induced parturition and abortion. Additionally, we detailed a range of angling practices and equipment, including hook-type, hook removal and emersion (i.e. air exposure), as well as inter- and intra-specific factors, including aerobic scope, respiratory mode, body size and species-specific behaviours, which are likely to influence injury and/or mortality rates and should therefore be considered when assessing angling-related impacts. We then utilized these data to provide a range of actionable recommendations for both anglers and policymakers which would serve to reduce the population-level impact of recreational angling on these enigmatic animals.

## Teaser

A summary of research on the impact of recreational catch-and-release angling for elasmobranchs, covering biochemical disruption- and physical injury-related impacts, and reporting associated mortality rates and sub-lethal consequences. Research findings are used to provide recommendations to policy makers and the angling community on how to enhance angling sustainability.

## Introduction

Commercial fisheries are traditionally considered to be the main global threat to elasmobranch populations (e.g. Field *et al.*, 2009; Campana, 2016; Dulvy *et al.*, 2021), but there is now a growing appreciation of the potential impacts of recreational angling for these species (e.g. Gallagher *et al.*, 2017*a*). In some cases, recreational shark catches may even exceed commercial ones (NOAA, 2014), with more than 66 million sharks caught by recreational anglers between 2005 and 2015 along the U.S. Atlantic coast alone, including 1.2 million individuals of prohibited species (Kilfoil *et al.*, 2017). Globally, elasmobranchs are estimated to represent approximately 5% to 6% of marine recreational angling catches (Freire *et al.*, 2020). Given the growing appreciation of the recreational angling sector’s potential impact, in recent years there has been a steady regulatory push towards catch-and-release fishing within the recreational angling community (e.g. Kneebone *et al.*, 2013), in many cases accompanied, or preceded, by a voluntary shift towards such methods by anglers (Gallagher *et al.*, 2017*a*).

When considering the negative impacts of angling it should be noted that many anglers hold conservation-oriented views (Shiffman *et al.*, 2017) and may participate in research and conservation activities (e.g. Drake *et al.*, 2005; Neat *et al.*, 2015). Additionally, anglers often fund conservation and research efforts either directly or indirectly (e.g. Barnett *et al.*, 2016; Cooke *et al.*, 2016) and, as noted previously, may voluntarily participate in catch-and-release angling, often in conjunction with practices believed to best promote animal welfare (Gallagher *et al.*, 2017*a*). However, it should be stated that even widespread application of catch-and-release angling does not equate to an absence of negative effects as, even under ideal conditions, a proportion of fish could be expected to die from biochemical disruptions and/or injuries related to fishing (Cooke and Schramm, 2007). Critically, while the impacts of catch-and-release angling are well documented for many recreationally important teleosts, such as salmonids (Cooke *et al.*, 2013) and cod (Weltersbach and Strehlow, 2013), these have only more recently come into focus for elasmobranchs and hence there are still important knowledge gaps which remain unresolved.

Recently an increasing number of studies have focussed on the conservation and animal welfare implications of catch-and-release activities within recreational angling fisheries where elasmobranchs are either the primary target or are commonly caught. Studies to date have primarily been centred around classifying the nature and severity of the associated injuries, biochemical disruptions and subsequent post-release behavioural responses in various elasmobranch species to catch-and-release fishing (e.g. Kneebone *et al.*, 2013; Lavender *et al.*, 2022; Shea *et al.*, 2022), alongside the associated mortality risks (e.g. Kneebone *et al.*, 2013; Sepulveda *et al.*, 2015; Anderson *et al.*, 2021). Several studies have also investigated the effects of specific angler behaviours or fishing equipment under the overarching umbrella of best practice catch-and-release, with the core aim of reducing recreational angling-related impacts (e.g. French *et al.*, 2015*a*; Mohan *et al.*, 2020; Weber *et al.*, 2021).

Here we attempt to synthesize research to-date on the various impacts of catch-and-release fishing for elasmobranchs, along with the associated mortality risks and sub-lethal consequences of such impacts, with an additional focus on the development of evidence-based best practice guidelines for the capture and release of these species. We provide a broad overview on the current state of research, highlighting current limitations of such research and key knowledge gaps, alongside recommendations for both future avenues of research and angling practices.

## Materials and Methods

Literature searches were carried out using Google scholar and within the Wiley, Elsevier and Springer online research databases using the search terms outlined in Table 1, with all possible search term combinations tested. The first 200 results from each search were individually assessed for their relevance to the research topic and included where relevant. In some searches, due to overlap with unrelated research areas, it was also necessary to exclude additional terms to better refine search results. For any studies which were of primary relevance (i.e. those which directly addressed impacts of catch-and-release angling), both the cited material within these and any subsequent literature citing the original study were also reviewed for their relevance. This approach was considered to give the greatest likelihood of identifying publications directly addressing the main research area, as well as those which may have only provided relevant information incidentally.

**Table 1:** Search terms used in literature search for peer-reviewed material using Google scholar and within the Wiley, Elsevier and Springer online research databases

Search Term Group 1	Search Term Group 2	Search Term Group 3
Catch and release	“Shark”	(none)
Post release	“Ray”	“Surviv*”
Capture release	“Skate”	“Mortality”
	“Elasmobranch”	“Stress”
		“Best practice”
		“Guideline”
		“Hypoxia”
		“Parturition”
		“Abortion”
		“Sublethal”
		“Physiolog*”
		“Reflex”
		“Behaviour”

## Results and Discussion

### Overview of Catch-and-Release Science

**Figure 1:**
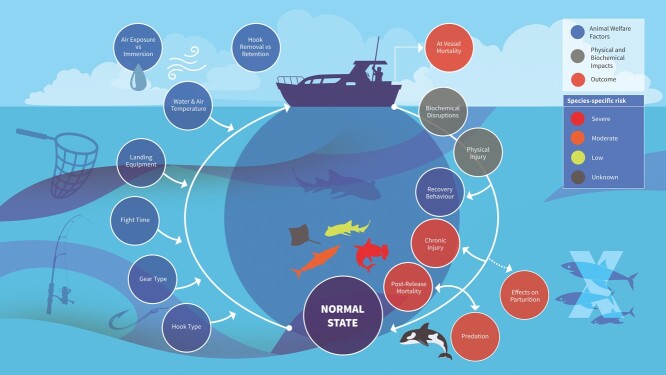
Diagrammatic representation of the major animal welfare factors; resultant physical and biochemical impacts; and outcomes associated with recreational catch-and-release angling for elasmobranchs. Species-specific risk categories are outlined for a nurse shark (*Ginglymostoma cirratum*; low); a ‘typical’ lamnid shark (family Lamnidae; moderate); a great hammerhead (*Sphyrna mokarran*; severe); and for a ‘typical’ rajid (family Rajidae; unknown).

A range of animal welfare implications are inevitably associated with recreational catch-and-release angling, given the inherent injury and stress associated with the process of capture via hook and line, handling (often including removal from the water and exposure to air) and release (Figure 1). These impacts can be broadly categorized as resulting from the physical injury and/or the biochemical disruptions associated with this process, the ultimate consequences of which may range from relatively minor short-term impairments to mortality (both direct and indirect).

Broadly in line with the above, the methods investigated in most catch-and-release studies can be classified into three groups:

1. physical injury/condition studies, in some cases also including assessments of reflex impairment.
2. biochemical studies, often involving measurements of blood parameters at either the point of capture or release
3. movement studies, which generally seek to assess behavioural recovery periods or post-release mortality rates based on data-logging or telemetry devices.

It is important to note that several studies have combined these methods, with combinations of blood parameters, reflex impairments and/or physical condition used as predictive measures for post-release mortality.

### Catch-and-release impacts

#### Physical injury-related impacts

Perhaps the most obvious impact of any form of hook and line fishing is the injury caused by the fishing hook(s) to the animal (Figure 1). These injuries can vary in severity from superficial injuries when animals are hooked in the outer portions of the mouth (Begue *et al.*, 2020; Keller *et al.*, 2020, 2021), to severe injuries caused by gill-hooking (French *et al.*, 2015*a*); internal ‘gut-hooking’ (Borucinska *et al.*, 2002, 2001; Kilfoil *et al.*, 2017); or tail hooking (Heberer *et al.*, 2010; Sepulveda *et al.*, 2015). Such events may also increase the difficulty of hook removal, leading to retention of fishing hooks post-release (Kilfoil *et al.*, 2017). Furthermore, the fishing line or leader material itself may also cause abrasions pre-capture and, where hooks are retained, post-release (Whitney *et al.*, 2017; Anderson *et al.*, 2021). Additionally, the processes and equipment used to on board (boat angling) or land (shore angling) elasmobranchs may cause injury. Animals are often lifted out of the water onto boats. Larger fish may sometimes be lifted by the gill slits or fins, or using gaffs, with the latter in particular presenting a risk of severe injury (Musyl and Gilman, 2018). Alternative equipment, such as landing nets used to lift elasmobranchs from the water, or ‘tail ropes’ used to secure sharks boatside by the caudal peduncle, have the potential to cause more minor injuries, such as skin abrasions (Bansemer and Bennett, 2010). Similarly, shore anglers may cause injuries by dragging elasmobranchs up beaches, (Shiffman *et al.*, 2017; Mohan *et al.*, 2020; Shiffman, 2020; Weber *et al.*, 2020; Binstock *et al.*, 2023). Finally, a factor which has received substantial attention in teleosts, but is far less studied in elasmobranchs, is the depth change (and associated pressure and temperature changes) to which fish may be subjected during capture. While elasmobranchs lack a swim bladder, there is evidence to suggest that capture from greater depths in commercial fisheries may still cause barotrauma related injuries (depth range: 746–1913 m; Endicott and Agnew, 2004; 900–1100 m; Rodríguez-Cabello and Sánchez, 2017). Additionally, elasmobranchs may also be exposed to rapid and substantial changes in water temperature (potentially > 10°C; Fiedler, 2010) when retrieved from mesopelagic waters (200–1000 m depth), passing through the thermocline, to the sea surface. However, such extreme depth ranges may only be relevant to specialized deep water anglers, with similar studies from more representative depth ranges for recreational angling currently lacking. Given the wide range of potential injuries associated with various fishing practices within both commercial fisheries and the recreational angling sector, physical condition scores have often been used to characterize the overall physical status of elasmobranchs at the point of capture (e.g. Gurshin and Szedlmayer, 2004; Whitney *et al.*, 2017; Musyl and Gilman, 2018). Physical condition is usually scored on a basic level from ‘healthy’ to moribund or dead animals (Laptikhovsky, 2004; French *et al.*, 2015*a*; Hyatt *et al.*, 2016), while also frequently including several categories for injury severity (Gurshin and Szedlmayer, 2004; Campana *et al.*, 2009). It is important to note that such scores are both qualitative and subjective and hence may be subject to individual bias (e.g. Campana *et al.*, 2009).

#### Biochemical-related impacts

While less easily observed than physical injuries, recreational angling can induce a range of biochemical disruptions in elasmobranchs (Figure 1), as shown by changes in concentrations of various biochemical indicators (e.g. Danylchuk *et al.*, 2014; Whitney *et al.*, 2017; Weber *et al.*, 2021). Many of these disruptions may stem from the energetic demands associated with the process of capture and/or hypoxemia induced by emersion (air exposure) during handling. However, marked increases in body temperature during emersion have also been documented (Wosnick *et al.*, 2018; Harding *et al.*, 2022), while increases in body temperature may also occur, at least in regionally endothermic shark species, due to extreme activity levels during capture (Otway, 2020). Although currently underexplored, capture from greater depths may also cause body temperature increases, given water temperatures at the surface generally exceed those at greater depths (Rodríguez-Cabello and Sánchez, 2017; Prohaska *et al.*, 2021). Such temperature changes have the potential to induce biochemical disruptions, including changes in the expression of heat-shock proteins (Weber *et al.*, 2021).

Most of the biochemical parameters studied have been those which can be readily obtained from blood samples. Of these, the majority are also directly linked to energetic demands, including blood glucose (e.g. Danylchuk *et al.*, 2014; French *et al.*, 2015*a*; Prohaska *et al.*, 2018), haematocrit (e.g. Hight *et al.*, 2007; Brill *et al.*, 2008; Heberer *et al.*, 2010) and catecholamine (Hight *et al.*, 2007) concentrations. Some parameters are more specifically linked to blood acidosis, caused by anaerobic respiration, including blood lactate, pCO_2_ and pH (e.g. Kneebone *et al.*, 2013; Whitney *et al.*, 2017; Weber *et al.*, 2021). Metabolic stress (oxidative and/or thermal), has also been assessed via erythrocyte heat shock protein (HSP) levels (e.g. Heberer *et al.*, 2010; French *et al.*, 2015*a*; Weber *et al.*, 2021). Additionally, blood concentrations of several inorganic salt ions (e.g. Mg^2+^, Ca^2+^, Na^+^, K^+^), which may be linked to numerous physiological processes, including acid–base regulation and ion transport, have also been recorded in several studies (e.g. Heberer *et al.*, 2010; Danylchuk *et al.*, 2014; Scarponi *et al.*, 2021). Furthermore, Guida *et al.* (2016) categorized overall energy balance using adenylate energy charge (AEC) within tissue samples. A single study also noted exertional rhabdomyolysis, or the breakdown of muscle tissue, causing release of muscle fibre contents into the blood, alongside acute renal failure, in shortfin mako sharks (*Isurus oxyrinchus*) subjected to sustained angling bouts (Otway, 2020). The mechanisms behind many of these parameters are extensively detailed in Skomal and Mandelman (2012) and Renshaw *et al.* (2012).

#### Biochemical proxies

In an attempt to produce a more readily assessed and less-invasive range of proxies for biochemical disruption (i.e. not requiring blood or tissue samples) a growing body of research has focussed on the use of reflex impairment indicators and/or assessments of specific behaviours, such as spiracular movement/buccal pumping and muscle tension (e.g. Gurshin and Szedlmayer, 2004; Laptikhovsky, 2004; Jerome *et al.*, 2018). Such assessments often form individual components within overall scores, such as ‘behavioural release condition scores’ (BRCS) or ‘reflex action mortality predictor’ (RAMP) scores (Danylchuk *et al.*, 2014; Hyatt *et al.*, 2016). This approach has been broadly applied to teleosts (e.g. Davis, 2010; Brownscombe *et al.*, 2017) and is increasingly being applied to elasmobranchs, either to predict post-release outcomes (Danylchuk *et al.*, 2014) or as an indicator for biochemical disturbances (Mannheim *et al.*, 2018). Reflex impairment assessments typically involve assessment of an individual’s responses to specific external stimuli, including the functioning of the nictitating membrane response, bite reflex and self-righting reflex (Danylchuk *et al.*, 2014; Gallagher *et al.*, 2014; Jerome *et al.*, 2018; Whitney *et al.*, 2021). This approach has shown promise in some studies, although to date results are still mixed, with the direct link between biochemical disruptions and subsequent reflex impairments not clear (Danylchuk *et al.*, 2014; Gallagher *et al.*, 2014; Jerome *et al.*, 2018).

### Catch-and-release outcomes

#### Post-release mortality

Mortality may be induced by angling either directly (e.g. Kneebone *et al.*, 2013; Danylchuk *et al.*, 2014; Sepulveda *et al.*, 2015), or indirectly, for example due to increased predation risks (e.g. Mohan *et al.*, 2020; Whitney *et al.*, 2021) (Figure 1). Sub-lethal consequences may be induced, for example, by reduced feeding ability, energetic costs of injury healing and/or secondary infections (e.g. Borucinska *et al.*, 2002, 2001). Although such impacts may in-turn lead to delayed or indirect mortality (Adams *et al.*, 2015; Kilfoil *et al.*, 2017), for the purposes of this section we included only documented mortalities (both direct and indirect). Mortalities are usually identified via data-logging or telemetry devices (e.g. Moyes *et al.*, 2006; Kneebone *et al.*, 2013; French *et al.*, 2015*a*; Whitney *et al.*, 2017; Mohan *et al.*, 2020). Post-release mortality is generally characterized based upon a lack of horizontal movement (Gurshin and Szedlmayer, 2004; Mohan *et al.*, 2020), lack of transmissions (Gallagher *et al.*, 2014) and/or static depth profiles, where an animal is assumed to be dead and resting on the seabed based on bathymetric data (Sepulveda *et al.*, 2015; French *et al.*, 2015*a*; Whitney *et al.*, 2016, 2017; Mohan *et al.*, 2020). In other cases mortality is inferred by tag detachment at a set maximum depth (e.g. Sepulveda *et al.*, 2015; Domingo *et al.*, 2018), sometimes corroborated by this exceeding the maximum recorded depth for that species (French *et al.*, 2015*a*).

Post-release mortality rates associated with recreational catch-and-release angling (excluding deliberately caudal fin-hooked common thresher sharks, *Alopias vulpinus*) were identified in 14 studies, representing a combined total of 412 elasmobranch individuals from 12 species captured, with all being either Lamniform (n = 152) or Carcharhiniform (n = 260) sharks (Table 2). These ranged from 0% (5 studies with no mortalities) to as high as 100% (Binstock *et al.*, 2023), with a mean value from all studies of 14.3% (i.e. 59 total post-release mortalities; Table 2). Overall post-release mortality rates were similar across both orders (Lamniformes = 13.8%; Carcharhiniformes = 14.6%), although the figure for Lamniformes was heavily skewed by the results of Kneebone *et al.* (2013), where juvenile sand tigers (*Carcharias taurus*) were subject to high levels of gut-hooking (42%). When this study was excluded the mortality rate for order Lamniformes was reduced to 5.7%. Similarly, great hammerheads (*Sphyrna mokarran*) showed by far the highest mortality rates within the Carcharhiniformes (100%; Table 2), albeit this was based upon a very small sample size (N = 2; Binstock *et al.*, 2023). Also note that this basic comparison does not account for inter-study differences in various risk factors such as emersion, hook removal, etc.

**Table 2:** Summary of studies including post-release mortality rates by species and order (^*^ mortalities from Sepulveda *et al.* (2015) are reported inclusive of mouth-hooked sharks only, i.e. excluding caudal fin-hooked sharks; ^**^For one of the individuals the suspected mortality occurred 14 days post-capture and could not be reliably confirmed, however the pattern of behaviour was considered indicative of mortality)

Study	Species	Order	Sample size (n)	Post-release mortalities (n)	Post-release mortality rate (%)
Sepulveda *et al.* (2015) ^*^	*Alopias vulpinus*	Lamniformes	7	0	0
Holts and Bedford (1993)	*Isurus oxyrinchus*	Lamniformes	3	0	0
French *et al.* (2015a)	*Isurus oxyrinchus*	Lamniformes	30	3	10
Anderson *et al.* (2021)	*Lamna nasus*	Lamniformes	14	0	0
Kneebone *et al.* (2013)	*Carcharias taurus*	Lamniformes	65	16	24.6
Kilfoil *et al.* (2017)	*Carcharias taurus*	Lamniformes	33	2	6.1
Gurshin and Szedlmayer (2004)	*Rhizoprionodon terraenovae*	Carcharhiniformes	10	1	10
Skomal and Chase (2002)	*Prionace glauca*	Carcharhiniformes	4	0	0
Danylchuk *et al.* (2014)	*Negaprion brevirostris*	Carcharhiniformes	32	4	12.5
Whitney *et al.* (2017)	*Carcharhinus limbatus*	Carcharhiniformes	31	3	9.7
Mohan *et al.* (2020)	*Carcharhinus limbatus*	Carcharhiniformes	22	5	22.7
Weber *et al.* (2020)	*Carcharhinus limbatus*	Carcharhiniformes	81	15	18.5
Binstock *et al.* (2023)	*Carcharhinus limbatus*	Carcharhiniformes	11	5	45.5
Binstock *et al.* (2023)	*Carcharhinus leucas*	Carcharhiniformes	14	0	0
Knotek *et al.* (2022)	*Carcharhinus acronotus*	Carcharhiniformes	47	3	6.4
Binstock *et al.* (2023)	*Galeocerdo cuvier*	Carcharhiniformes	6	0	0
Binstock *et al.* (2023)	*Sphyrna mokarran*	Carcharhiniformes	2	2^**^	100
Lamniformes total			152	21	13.8
Carcharhiniformes total			260	38	14.6
Total			412	59	14.3

All studies reporting mortalities found that the majority occurred in the initial 5 days post-release (Sepulveda *et al.*, 2015) and usually within hours (e.g. Gurshin and Szedlmayer, 2004; French *et al.*, 2015*a*; Whitney *et al.*, 2016, 2017). Mortalities were generally typified by inactive or moribund behaviour, with sharks either resting on the bottom or gradually sinking through the water column prior to tag detachment at a set depth (Whitney *et al.*, 2016; Mohan *et al.*, 2020). In some cases this was interrupted by brief periods of sporadic activity before cessation of all activity (Whitmore *et al.*, 2016). The timing of this behaviour varied from immediately post-release (e.g. Heberer *et al.*, 2010; Mohan *et al.*, 2020); to minutes (e.g. Gurshin and Szedlmayer, 2004; Danylchuk *et al.*, 2014; Whitney *et al.*, 2017); hours (e.g. Heberer *et al.*, 2010; French *et al.*, 2015*a*; Whitney *et al.*, 2016, 2017); or days post-release (e.g. Sepulveda *et al.*, 2015; Kilfoil *et al.*, 2017).

Of the physical injury-related impacts, the most commonly identified cause for post-release mortality is physical injury caused by fishing hooks, either through hook retention, generally associated with gut-hooking (Adams *et al.*, 2015; Kilfoil *et al.*, 2017), or through excessive bleeding, generally caused by injuries to the gills (Gurshin and Szedlmayer, 2004; French *et al.*, 2015*a*; French, 2017). As previously noted, barotrauma injuries have been recorded in commercial fisheries, with longline capture from greater depths linked to reduced survival rates of *Raja* sp. anon (depth range: 746–1913 m; Endicott and Agnew, 2004) and deep water shark species (depth range 900–1100 m; Rodríguez-Cabello and Sánchez, 2017). However, the relevance to recreational angling at shallower depths is unknown. Conversely, angling from the relatively shallower depths associated with shore angling may also pose greater mortality risks (Weber *et al.*, 2020; Binstock *et al.*, 2023), with these potentially linked to injuries associated with lifting or dragging of animals out of the water and onto beaches, jetties, rocks or piers and/or to greater biochemical disruptions stemming from longer air exposure periods or prolonged restraint in relatively warm and shallow coastal waters.

As might be expected, moribund health scores and severe injuries have mostly been found to represent strong predictors for subsequent post-release mortality (Campana *et al.*, 2016, 2009; Musyl and Gilman, 2019, 2018), although not in all cases (French *et al.*, 2015*a*; Domingo *et al.*, 2018). Less severe categories are generally poorer predictors (Hyatt *et al.*, 2016; Domingo *et al.*, 2018; Anderson *et al.*, 2021), although with some exceptions (Musyl and Gilman, 2019, 2018). Notably, barring a few exceptions (e.g. Gurshin and Szedlmayer, 2004; Whitney *et al.*, 2017), most such studies have been performed within commercial longline fisheries (e.g. Campana *et al.*, 2009; Musyl and Gilman, 2018; Bowlby *et al.*, 2020), where injuries, typically under industrial, harvest-oriented settings, are often more severe than in recreational angling settings. Thus, the more severe categories (and best predictors of mortality) may have limited relevance to recreational angling. Additionally, the high subjectivity of such observations can mean that variability in categorization among observers may overwhelm any biological differences (Campana *et al.*, 2009).

Of the numerous biochemical measures studied, the most consistently reliable predictor of mortality has been blood lactate concentration (e.g. Moyes *et al.*, 2006; Renshaw *et al.*, 2012; Whitney *et al.*, 2021), although this may partly relate to the relative abundance of studies recording lactate levels. This pattern likely relates to the availability of portable blood analysers (e.g. i-STAT, Lactate Pro), initially designed for human use, which have been adopted as rapid and relatively accurate ways to determine blood pH and lactate levels (Awruch *et al.*, 2011; Harter *et al.*, 2015). Nevertheless, in studies where multiple haematological variables have been measured, blood lactate has consistently emerged as among the strongest predictors of post-release survival/mortality (Moyes *et al.*, 2006; Whitney *et al.*, 2021).

#### Post-release recovery behaviour

Aside from directly causing mortality, biochemical disruptions and/or physical injuries may also lead to downstream behavioural impacts (Figure 1), including specific post-release recovery behaviours (e.g. Bullock *et al.*, 2015; Whitney *et al.*, 2017; Lavender *et al.*, 2022). These may include periods of increased or decreased activity, changes in depth usage patterns or differences in tailbeat frequency (Gurshin and Szedlmayer, 2004; Whitney *et al.*, 2017, 2016; Anderson *et al.*, 2021; Lavender *et al.*, 2022). Some studies have hypothesized that these behaviours may relate to excess post-exercise oxygen consumption (EPOC) caused by respiratory and metabolic acidosis (Bouyoucos *et al.*, 2019; Weber *et al.*, 2021; Iosilevskii *et al.*, 2022) and/or to elevated catecholamine levels (Skomal, 2006; Weber *et al.*, 2021). The reported duration of these behaviours varies widely, with values ranging from < 1 hour (Gurshin and Szedlmayer, 2004; Bullock *et al.*, 2015), to ≤ 10 hours (Spargo, 2001; Whitney *et al.*, 2017, 2016), to ~ 1 day (French *et al.*, 2015*a*; Whitney *et al.*, 2016, 2017) and even several days (Anderson *et al.*, 2021), closely matching the timing of most post-release mortalities within individual studies. Post-release recovery behaviours may lead to indirect mortality through increased predation risks (Sepulveda *et al.*, 2019; Mohan *et al.*, 2020; Whitney *et al.*, 2021, 2017), although it may be difficult to determine whether predation is related to the initial capture event (Sepulveda *et al.*, 2019; Whitney *et al.*, 2021). Such predation events are typified by a period of constant light levels recorded by the attached data-logging package (Mohan *et al.*, 2020), a change in temperature (Sepulveda *et al.*, 2019; Mohan *et al.*, 2020) and/or changes in activity patterns (Sepulveda *et al.*, 2019; Whitney *et al.*, 2021, 2017).

#### Chronic health impacts

Although not subject to extensive research and, hence, generally only reported incidentally, some long-term chronic impacts have been reported for elasmobranchs subjected to both recreational and commercial hook and line fishing, with almost all of these linked to hook retention (Figure 1). In this instance comparisons can be cautiously made between recreational angling and commercial fisheries, for which a much greater body of research exists, given the impacts of retained hooks are likely to be similar, irrespective of the specific fishing method (although less animal welfare conscious attempts to remove hooks in commercial fisheries may cause more severe injuries, even where hook removal is unsuccessful; Bansemer and Bennett, 2010; Campana *et al.*, 2016). Several studies on post-release survival of both recreationally and commercially caught elasmobranchs have reported which individuals had retained hooks, with some then correlating these with post-release mortality rates (e.g. Kneebone *et al.*, 2013; French *et al.*, 2015*b*; Kilfoil *et al.*, 2017). Borucinska *et al.* (2002) noted that retained hooks identified in 6 captured blue sharks (*Prionace glauca*) led to severe and chronic health problems, including damage to various internal organs, in all cases, while Mucientes and Queiroz (2019) reported severe damage to the oesophagus of a shortfin mako shark due to a retained hook. Adams *et al.* (2015) also reported the cause of death for a shortfin mako shark as being due to a retained hook, with Kilfoil *et al.* (2017) reporting the same for a sand tiger.

#### Effects on parturition

An additional impact of both recreational and commercial fishing for elasmobranchs, which has received surprisingly little attention, and for which the specific cause(s) and physiological mechanisms remain unknown, is the effect of capture on parturition. Capture-induced parturition, or abortion, has been directly reported for at least 89 live young bearing elasmobranch species within 27 families, (Adams *et al.*, 2018; Wosnick *et al.*, 2019). Although most of these examples came from commercial fisheries (Adams *et al.*, 2018; Wosnick *et al.*, 2019), several miscellaneous reports and social media posts indicate that capture-induced parturition/abortion also occurs as a result of recreational angling (Adams *et al.*, 2018). Adams *et al.* (2018) also cautioned that the current scarcity of such reports may be due to parturition being unobserved, rather than infrequent, as parturition may occur prior to landing or post-release. Both Wosnick *et al.* (2019) and Adams *et al.* (2018) noted that neonates rarely, if ever, survived, irrespective of the development stage, following capture-induced parturition. Thus, this phenomenon may represent an important cryptic effect of recreational catch-and-release angling, particularly if pregnant females represent a substantial proportion of the catch. Furthermore, although the relevance to recreational angling is unknown, capture of pregnant elasmobranchs in trawls may lead to other negative effects on parturition, including reduced neonate size, as observed for southern fiddler rays (*Trygonorrhina dumerilii*) (Guida *et al.*, 2017).

### Animal Welfare Factors—Implications for best practice

#### General (handling & equipment)

Likely the most studied determining factors for fisheries impacts in both commercial fisheries and recreational angling are the choice of hook-type and the removal or non-removal of fishing hooks at the point of capture, with these two factors often intrinsically linked. Although there are a multitude of different ‘hook-types’ available to recreational anglers, these can usually be simplified to one of the two most common hook-types; the traditional j-hook and the circle-hook, so named due to their profile. Additionally, some circle-hook designs may have a hook point which is at an offset angle, rather than in line with the hook shank, with some anglers preferring these due to a perceived higher catch rate. While most fishing hooks also include a barb after the hook point, ‘barbless’ hooks are also available, although the use of barbed or barbless hooks has received surprisingly little attention within the field of elasmobranch research, despite evidence that they can reduce unhooking times and aid in hook removal for teleosts (reviewed in Cooke and Sneddon, 2007).

Across all fishing methods, most studies have indicated a reduced risk of gut-hooking and increased chance of jaw-hooking with the usage of circle-hooks (Afonso *et al.*, 2011; Pacheco *et al.*, 2011; French *et al.*, 2015*a*; French, 2017; Keller *et al.*, 2020), as well as a lower risk of foul-hooking (i.e. hooking outside the mouth), which has also been linked to mortality (Heberer *et al.*, 2010; French *et al.*, 2015*a*; French, 2017). These findings are further supported by meta-analyses of commercial longline data (Godin *et al.*, 2012; Reinhardt *et al.*, 2018; Keller *et al.*, 2021). Reinhardt *et al.* (2018) noted lower at-vessel mortality for 3 shark species (oceanic whitetip, *Carcharhinus longimanus*; shortfin mako, *Isurus oxyrinchus*; and scalloped hammerhead, *Sphyrna lewini*) when circle-hooks were used, while Godin *et al.* (2012) concluded that, overall, use of circle-hooks corresponded with a significant decrease in gut-hooking and at-vessel-mortality. However, when considering the conservation benefits of circle-hook usage, some cautionary points should be noted. Firstly, offset point circle-hooks may cause higher gut-hooking rates than non-offset circle-hooks (Rice *et al.*, 2012). Additionally, retained circle-hooks may take longer for some species to shed compared to j-hooks, with one study noting that retained j-hooks were shed by pelagic stingray (*Pteroplatytrygon violacea*) within 6 days (n = 6), while circle-hooks took an average of 44.5 days to be shed (n = 4) and up to 125 days (François *et al.*, 2019).

Similarly to hook-type, the practices of either removing hooks or cutting the line or wire leader material above hooks (i.e. causing hook retention) has also been relatively well-studied. As previously noted, retained hooks may cause severe and chronic health effects (Borucinska *et al.*, 2002, 2001; Adams *et al.*, 2015), while trailing fishing line may also have negative effects, such as tissue necrosis (François *et al.*, 2019). However, studies on teleosts indicate that, in some cases, hook removal may actually be detrimental to survival due to greater emersion periods (Cooke and Wilde, 2007; Wilde and Sawynok, 2009), with one study on post-release survival of longline-caught shortfin mako sharks indicating this may also be the case for some elasmobranch species (Domingo *et al.*, 2018). Hook removal from gut-hooked elasmobranchs may also be difficult or impossible without causing severe injury to the animal (Kilfoil *et al.*, 2017).

Where fishing hooks are retained by the animal post-release, hook location may be a critical factor in determining post-release outcomes, with hook retention in gut-hooked elasmobranchs linked to chronic and sometimes fatal health problems (Borucinska *et al.*, 2002, 2001; Lécu *et al.*, 2011; Adams *et al.*, 2015) and both increased short- and long-term mortality rates (Campana *et al.*, 2009; Kilfoil *et al.*, 2017). In contrast, where retained hooks are located in the outer part of the jaw, negative impacts such as tissue necrosis may be less severe, although still an issue in some cases (Bansemer and Bennett, 2010). Begue *et al.* (2020) noted that tiger sharks with (in some cases multiple) externally visible retained fishing hooks shed these within 6 months on average and appeared to be largely unaffected, in terms of feeding ability, growth, reproduction and tissue necrosis. Likewise, Chin *et al.* (2015) noted that blacktip reef sharks (*Carcharhinus melanopterus*) also appeared to be extremely resilient to visible retained fishing hooks.

Given the difficulties sometimes associated with hook removal in elasmobranchs, the choice of hook materials may be key to reducing chronic impacts. Corrodible hooks are more likely to be quickly shed, as shown by Begue *et al.* (2020), who compared 50% hook retention probabilities in tiger sharks for corrodible (5.7 months) and stainless steel (7.8 months) hooks. Indeed, use of corrodible hooks is mandated in some commercial longline fisheries (NOAA, 2003). However, Begue *et al.* (2020) speculated that, in some cases only the external portion of the hook may be lost, with the internal proportion remaining. Horst (2000) also speculatively hypothesized that corrosion of such hooks might lead to greater tissue damage, and thus lower survival, due to galvanic action. It should also be noted that all hooks will eventually corrode, but at different rates and with different lag times depending on the material thickness and type of anti-corrosive coating used.

Another factor which has received substantial attention is the time spent between hooking and landing a shark or ray, often termed as ‘fight time’, in some cases also reported inclusive of handling time, although these two capture stages involve different stressors and thus such studies should not be considered directly comparable (e.g. Skomal and Chase, 2002;Danylchuk *et al.*, 2014 ; Shea *et al.*, 2022). Fight time relates to a complex combination of angler and fish behaviour, fish size and energy status and fishing equipment, including line class and drag setting (McLoughlin and Eliason, 2008; Cooke *et al.*, 2013; Danylchuk *et al.*, 2014; French, 2017; Kilfoil *et al.*, 2017), as well as species-specific factors, with protracted fight times reported both anecdotally and experimentally for lamnids and great hammerheads (French *et al.*, 2015*a*; French, 2017; Anderson *et al.*, 2021). Thus, it may be difficult to disentangle these numerous interlinked factors, particularly given the small sample sizes often used in such studies (e.g. Danylchuk *et al.*, 2014; French *et al.*, 2015*a*; Shea *et al.*, 2022), potentially contributing to the null effects seen in some studies (Danylchuk *et al.*, 2014; Shea *et al.*, 2022). However, despite these difficulties, several studies have reported significant biochemical effects from increasing fight times (e.g. Kneebone *et al.*, 2013; French, 2017; Whitney *et al.*, 2017; Weber *et al.*, 2021), although no survival impacts have yet been documented.

Differences in emersion periods and comparison of air-exposed versus non-air-exposed elasmobranchs have been studied via both recreational angling and commercial fisheries, as well as experimental emersion studies. In general, study results have followed a pattern of increased biochemical disturbance, longer recovery times and/or decreased survival following emersion (Cicia *et al.*, 2012; Murray *et al.*, 2015; Bowlby *et al.*, 2020, 2021), or with longer emersion durations (Dicken *et al.*, 2006; Cicia *et al.*, 2012; Murray *et al.*, 2015), although Weber *et al.* (2021) found that emersion changed the primary mechanism of blood acidosis (i.e. respiratory vs. metabolic) but not the degree of acidosis experienced by rod-and-line caught blacktip sharks. It should be noted that emersion durations in lab and commercial fishery studies often far exceed what would be expected in a recreational angling setting (Laptikhovsky, 2004; Cicia *et al.*, 2012; Mandelman *et al.*, 2013; Lambert *et al.*, 2018). Even recreational angling-based studies may involve non-representative emersion periods due to the need to obtain measurements, biological samples and/or apply data-logging packages. Conversely, changes in angler behaviour in the presence of scientists or participation of the most conservation-minded anglers in such research may shorten emersion periods relative to standard angling practices.

Both water and air temperature appear to play a role in the severity of catch-and-release impacts. Higher water temperatures are well known to reduce survival in teleosts (reviewed in Gale *et al.*, 2013), while several studies have indicated that this may also hold true for elasmobranchs (French *et al.*, 2015*a*; Whitney *et al.*, 2017; Knotek *et al.*, 2022; Binstock *et al.*, 2023). The temperature change associated with emersion may also have an effect, with greater disparity between water and air temperatures shown to cause changes in elasmobranch surface body temperature, which may be greater in smaller individuals (Wosnick *et al.*, 2018; Harding *et al.*, 2022), and with acute thermal shock during emersion also shown to induce additional biochemical disturbances (Cicia *et al.*, 2012).

Surprisingly little research attention has been paid towards the landing and handling practices for elasmobranchs, as well as the usage of various types of assisting equipment to on board or land them or secure them at the boat side to enable, for example, hook removal, measurement, tagging and/or photography. This is despite the injury risks associated with equipment, such as gaffs (Musyl and Gilman, 2018) and tail ropes (Bansemer and Bennett, 2010), and with practices, such as dragging sharks up beaches (Shiffman *et al.*, 2017), or misplacement of body ropes around the head and gill area (Mohan *et al.*, 2020). The lack of such studies may relate to the relatively recent timing of most catch-and-release studies, with usage of gaffs in particular increasingly rare among some groups of anglers practicing catch-and-release (French, 2017; French *et al.*, 2019*b*). However, gaffs may still be used in some angling communities, even where elasmobranchs are frequently released (Dicken *et al.*, 2006; Lynch *et al.*, 2010; French *et al.*, 2019*b*). Similarly, other related factors, such as specific handling methods for shore and boat anglers, or for dealing with more vulnerable species or pregnant females, have received little or no research attention, despite many angler best practice guides covering these topics (Authors, pers. obs.).

#### Inter and Intra-specific risk factors

Several studies have sought to quantify inter- and intra-specific differences in the biochemical response and/or survival rates from both catch-and-release recreational angling (e.g. Skomal, 2006; Hight *et al.*, 2007; Heberer *et al.*, 2010; Scarponi *et al.*, 2021) and commercial fishing methods (e.g. Bowlby *et al.*, 2020; e.g. Campana *et al.*, 2016; Gallagher *et al.*, 2014; Hight *et al.*, 2007). Some studies have also investigated contributing factors to these differences, including specific hunting and feeding behaviours (Heberer *et al.*, 2010; Sepulveda *et al.*, 2019, 2015; Aalbers *et al.*, 2021), differences in respiratory mode (McLoughlin and Eliason, 2008; Dapp *et al.*, 2016; Aalbers *et al.*, 2021) and metabolic factors (Marshall *et al.*, 2012; Skomal and Mandelman, 2012).

It has been hypothesized by several authors that a key factor influencing mortality rates from all methods of hook and line capture may be the relationship between a species’ behavioural response to capture and its aerobic scope, defined as the difference between minimum and maximum oxygen consumption rates (Clark *et al.*, 2013), with a greater aerobic scope leading to reduced blood acidosis and thus less severe post-release effects (e.g. Marshall *et al.*, 2012; Skomal and Mandelman, 2012; French *et al.*, 2015*b*). There is limited information available on the metabolic rates for most elasmobranchs; however, lamnid sharks are often hypothesized to possess a higher aerobic scope than carcharhinids, owing to regional endothermy (Marshall *et al.*, 2012; French *et al.*, 2015*b*). This has been hypothesized as being a major contributor to their relatively high post-release survival (French *et al.*, 2015*b*; Anderson *et al.*, 2021), despite often pronounced behavioural responses to capture (e.g. French *et al.*, 2015*b*; Otway, 2020; Anderson *et al.*, 2021). It should however be noted that the regional endothermy and prolonged fight times of some lamnids may contribute to biochemical disruptions not experienced by other elasmobranchs, including exertional rhabdomyolysis Otway (2020).

In contrast to the lamnids, carcharhinid sharks are hypothesized to possess a lower aerobic scope and may hence utilize anaerobic respiration to a greater extent during bouts of increased activity (Mandelman and Skomal, 2009), leading to greater biochemical disruption for a given activity level. The behavioural responses of carcharhinids to capture appear to vary widely, albeit much of the available data come from commercial fisheries so it is not clear how such patterns may translate to recreational angling. Longline and drumline based studies have recorded subdued behavioural responses in nurse sharks (*Ginglyostoma cirratum*) and tiger sharks (*Galeocerdo cuvier*) versus comparatively extreme responses in blacktip sharks (*Carcharhinus limbatus*) and great hammerheads (Mandelman and Skomal, 2009; Gallagher *et al.*, 2017*b*, 2014; Bouyoucos *et al.*, 2018). Correspondingly, great hammerheads and blacktip sharks caught using such methods have been found to be subject to severe biochemical disturbances and reflex impairments and very high at-vessel and/or post-release mortality (e.g. Mandelman and Skomal, 2009; Gallagher *et al.*, 2014; Jerome *et al.*, 2018), while sympatric tiger sharks have much lower mortality rates (Mandelman and Skomal, 2009; Gallagher *et al.*, 2014). The results of Binstock *et al.* (2023) also suggest a similar pattern in shore-based recreational fisheries, with blacktip and great hammerhead sharks subject to far higher post-release mortality rates than sympatric bull sharks (*Carcharhinus leucas*) or tiger sharks (Table 2). Furthermore, anecdotal reports from recreational anglers also support these observations regarding both the behavioural response to capture and poor post-release survival in great hammerheads (McClellan Press *et al.*, 2016).

The relative risk posed by hypoxemia during handling is also likely to vary greatly between species, based on respiratory mode, metabolic rate and hypoxemia tolerance, although this has received only limited attention (Cicia *et al.*, 2012), with comparative studies currently lacking. Hypoxemia may be more readily induced in ram-ventilating species compared to species which perform spiracle- or buccal pumping, potentially even without emersion, due to reduced water flow over the gills (McLoughlin and Eliason, 2008; Dapp *et al.*, 2016; Aalbers *et al.*, 2021). This may be an issue when sharks are restrained alongside a boat (e.g. Sepulveda *et al.*, 2015; Shea *et al.*, 2022) or held stationary in shallow water at beaches (e.g. Shiffman *et al.*, 2017; Hingley, 2020). There is evidence to suggest that capture-induced hypoxemia may be mitigated by pumping water over a shark’s gills (Davie *et al.*, 1993), albeit this study was carried out using sharks under tonic immobility. The outcomes of hypoxemia may not be immediate (i.e. at-vessel-mortality), with a potential for post-release effects such as respiratory acidosis, which may compound metabolic acidosis from struggling on the line (Weber *et al.*, 2021), resulting in post-release mortality or prolonged recovery periods (e.g. Renshaw *et al.*, 2012; Murray *et al.*, 2015; Bowlby *et al.*, 2020). Post-release mortality may also occur in ram-ventilating species if exhaustion prevents adequate gill ventilation (Dapp *et al.*, 2016).

Individual species or genera may have specific feeding or hunting behaviours which place them at a greater risk of severe recreational fishing-related impacts (Heberer *et al.*, 2010; Sepulveda *et al.*, 2019, 2015; Aalbers *et al.*, 2021). For example, thresher sharks (*Alopias* spp.) are often (both deliberately and accidentally) foul-hooked in the tail due to their specific feeding behaviour, where they use their elongated upper caudal lobe to stun prey (Heberer *et al.*, 2010; Sepulveda *et al.*, 2015). This can lead to increased mortality (up to 26%; Heberer *et al.*, 2010) as the sharks are unable to ram-ventilate when retrieved tail-first. In this case, usage of circle-hooks is extremely effective in preventing tail hooking, thereby reducing mortality rates (Heberer *et al.*, 2010; Sepulveda *et al.*, 2015). In contrast, the gulp feeding mechanism of sand tigers may result in high rates of gut-hooking, irrespective of the hook-type used (Kneebone *et al.*, 2013; Kilfoil *et al.*, 2017) and thus mitigation may be challenging or impossible.

The results of studies investigating the effects of inter- and intra-specific body size differences in a recreational angling setting have been mixed, with the body of research on this topic currently limited. A number of commercial longline-based studies have indicated that biochemical disturbances and at-vessel-mortality may decrease with increasing individual size (e.g. Diaz and Serafy, 2005; Braccini and Waltrick, 2019; Bowlby *et al.*, 2021). However, recreational angling-based studies have reported both concurrent (Whitney *et al.*, 2017; Shea *et al.*, 2022) and contrasting results (Heberer *et al.*, 2010; Scarponi *et al.*, 2021). In species which exhibit pronounced behavioural responses to capture, larger individuals may experience prolonged fight times, leading to greater biochemical disruption (Heberer *et al.*, 2010). Alternatively, the relatively greater glycogen stores of larger individuals may lead to lower biochemical disruption (Jerome *et al.*, 2018), with this potentially offsetting any additional energy expenditure in species where behavioural responses to capture are less pronounced.

### Current research limitations and knowledge gaps

Catch-and-release science has become an increasingly hot topic within elasmobranch research, as evidenced by a marked increase in the publication rate of such studies in the last 10 years. However, much of the current research has focussed on the orders Carcharhiniformes (23 catch-and-release studies identified) and Lamniformes (12 studies). In contrast, we identified relatively few studies on the orders Rajiformes (4 studies), Rhinopristiformes (3 studies) and Orectobliformes (1 study). Other elasmobranch orders, including Squaliformes and Squatiniformes, were not found to be represented by any catch-and-release studies. Even within the two most studied orders most research has focussed on only a handful of commonly targeted species. The lack of studies on rajids in particular may have important conservation implications, given many rajid species are commonly targeted by recreational anglers. Currently most knowledge on capture-related effects for rajids comes from commercial trawling studies, where fishing and handling practices differ greatly from recreational angling (e.g. Laptikhovsky, 2004; Arlinghaus *et al.*, 2007; Enever *et al.*, 2009; Mandelman *et al.*, 2013). To our knowledge, only a single study has investigated post-release behavioural impacts relating to recreational fishing for rajids, with this study unable to assess post-release survival as data-logging packages had to be physically recovered from recaptured flapper skates (*Dipturus intermedius*) (Lavender *et al.*, 2022). From a management perspective this makes it impossible to accurately determine angling impacts where no species-, family-, or even order-specific mortality rates have been documented.

From a practical perspective, when choosing biochemical indicators to predict post-release outcomes, the current body of evidence strongly indicates that blood lactate concentration is the best predictor for post-release mortality or survival (e.g. Moyes *et al.*, 2006; Renshaw *et al.*, 2012; Whitney *et al.*, 2021) and carries the additional advantage of being relatively easy to assess using portable blood lactate analysers. However, the high degree of inter-specific variation in blood lactate concentrations, even between closely related or congeneric species (Marshall *et al.*, 2012; Renshaw *et al.*, 2012; Gallagher *et al.*, 2014), presents an issue. Hence, accurate prediction of post-release mortality would likely require determination of species-specific lactate mortality thresholds. Similarly, when using physical condition scores to characterize physical status of animals, differences in the weighting of health scores may hamper inter-study comparison. We suggest that consideration be given to adopting a standardized scoring system to minimize such issues.

Much research has been focussed on the short-term lethal consequences of catch-and-release angling, with various telemetry and/or data-logging device based studies providing a substantial body of research on the short—medium term (≲1 month) post-release mortality rates for a range of elasmobranch species (e.g. Danylchuk *et al.*, 2014; Sepulveda *et al.*, 2015; French *et al.*, 2015*a*). In contrast, there has been comparatively little research on long-term consequences of recreational angling, such as those stemming from hook retention. Beyond the obvious expense and difficulty associated with prolonged monitoring periods, any assessment of the wider population-level impact is also likely to be hampered by a lack of available information on the rates of hook retention for most recreational fisheries targeting elasmobranchs. Most reported hook retention rates come from experimental studies (e.g. Kneebone *et al.*, 2013; Danylchuk *et al.*, 2014; Kilfoil *et al.*, 2017), where researcher and/or volunteer angler behaviour may differ from that of typical recreational anglers, or from small subsets of specific angling communities where similar caveats may apply (Dicken *et al.*, 2006; McClellan Press *et al.*, 2016), meaning the overall risk posed by this behaviour is difficult to reliably quantify. This pattern also highlights a broader lack of information on angler behaviour, with studies to date covering only a small number of specific angling groups and species, and with angler behaviours varying widely between these (e.g. McClellan Press *et al.*, 2016; Shiffman *et al.*, 2017; French *et al.*, 2019*a*, 2019b). When combined with the lack of data on how angler behaviours such as hook removal affect mortality rates, this makes predicting the population-level impact of specific angler behaviours near-impossible.

### Considerations for best practice advice to anglers

While current understanding of many of the animal welfare considerations noted above is limited, and substantial knowledge gaps remain for many taxa, there is sufficient evidence from recreational angling studies, with some inferences made from commercial and ex-situ studies, to provide several key recommendations to the angling community.

#### Angling practices

When taken together, research findings show that gut-, gill- and foul-hooking result in higher mortality rates from both direct hooking injuries, and from either extended emersion periods and unhooking injuries where hooks are removed, or from chronic health impacts if hooks are retained post-release. In comparison, jaw-hooking may ease unhooking (potentially eliminating the need for emersion), lead to fewer unhooking injuries and a higher likelihood of hook removal, and result in fewer negative impacts if hooks are retained. Therefore, the usage of non-offset, barbless circle-hooks and, where possible without causing further injury, removal of hooks before release, are key steps to reduce both lethal and chronic sub-lethal effects of recreational angling. Additionally, use of corrodible hooks may reduce the long-term impacts of retained hooks. Furthermore, the choice of landing equipment also has the potential to greatly impact injury risks for elasmobranchs. The use of gaffs should be avoided, given the potential for severe injury. Similarly, tail ropes should only be used where necessary and should ideally be constructed from a low friction rope material to minimize skin damage.

Beyond reducing physical injury, the most beneficial practices appear to be those which can reduce the level of respiratory or metabolic acidosis and other biochemical disturbances experienced by an animal during and after capture. Although recreational angling specific data are limited, there is clear evidence demonstrating that emersion is a significant stressor for elasmobranchs and should thus be minimized or eliminated if possible, to maximize survival likelihood. Likewise, irrespective of the difficulty in detecting such effects, there is evidence to suggest that reducing fight times will reduce mortality risks in elasmobranchs, with such benefits likely to be most easily achieved by use of appropriately rated fishing gear, including use of lines of relatively higher breaking strain, thus allowing increased resistance to be applied using reel drag settings. While the level of conservation benefit associated with these practices is likely to vary based on respiratory mode, aerobic scope and behavioural responses to capture, the overall pattern of reduced biochemical disturbance with reduced fight times and emersion appears to hold true across most, if not all, elasmobranch species studied. Furthermore, cognisance should be given to water and air temperatures during angling, with higher temperatures likely to induce greater biochemical disturbances, particularly where animals are removed from the water. Finally, specific animal welfare considerations should be made for species or genera which may carry a higher risk of post-release mortality, such as hammerheads, thresher sharks and sand tigers, and for pregnant females, given the potential for capture-induced effects on parturition.

#### Human dimension

Consideration of the potential impact of the various recommendations outlined above must consider the correlation (or lack thereof) between scientific research, angler guidance and real-world changes in angler behaviour. Best practice advice is only likely to have conservation benefits if paired with effective research dissemination and education/outreach programmes targeted towards the recreational angling community. A prime example of this can be seen in the United States, where anglers wishing to fish for sharks in US federal waters of the Atlantic, Caribbean and Gulf of Mexico must complete a short online training course to obtain the relevant federal permits, with this course outlining best practices for shark angling, as well as the identification of prohibited shark species which cannot be landed for conservation reasons. A major barrier to improving angler behaviour regarding animal welfare may be an overall lack of knowledge on the conservation status of many elasmobranch species within the angling community, as well as the belief that recreational angling has little to no impact on elasmobranch populations (Gallagher *et al.*, 2015; Shiffman *et al.*, 2017). Therefore, the decline in many elasmobranch populations must be articulated to anglers and their role in this identified, so that they can amend any unwitting and potentially injurious practices which may result from unfamiliarity about the conservation status of some species and the potential impact of their actions. Anglers can act responsibly when practicing catch-and-release, with case studies demonstrating high levels of willingness to implement conservation-minded behaviours in parts of Australia (Heard *et al.*, 2016). Therefore, successfully engaging with anglers and further alerting them to the risks posed by—and the likely benefits of avoiding—injurious practices is likely to have a tangible benefit in maximizing elasmobranch welfare and conservation outcomes.

As a case-study demonstrating the importance of angler engagement, one issue which must be addressed by scientists is the view among some anglers that circle-hooks reduce shark or ray catchability, with this likely to act as a key barrier to angler adoption (Serafy *et al.*, 2012). In contrast to this, numerous studies on longline fisheries and recreational angling have indicated that circle-hooks have either a null or non-significant effect on catch rates (Gulak and Carlson, 2021; Keller *et al.*, 2020; e.g. Yokota *et al.*, 2006), as backed up by meta-analyses of commercial data (Godin *et al.*, 2012; Keller *et al.*, 2021). A few studies have also reported significant positive effects (Afonso *et al.*, 2011; Foster *et al.*, 2012; Hannan *et al.*, 2013), while, to our knowledge, no studies have reported large negative impacts on catch rates. In a recreational angling context, Willey *et al.* (2016) reported higher hooking and capture rates with circle-hooks for 10 shark species. This perception among anglers therefore represents an inability by scientists and fisheries managers to effectively communicate this near-consensus of results to the angling community.

## Conclusions

Elasmobranch catch-and-release science is a rapidly growing research field, with continual advancements in our overall knowledge as new research findings are published. This rate of progress notwithstanding, there are several taxonomic groups which have received little to no research attention, despite many species within these being targets for anglers. Hence, there is a clear need to better match research effort to those species most likely to be impacted by the recreational angling sector. Additionally, better standardization of the variables recorded during catch-and-release studies, such as physical condition scores, may aid inter-study comparison. Such an approach may ultimately enable quantification of combined mortality risks, using the most reliable physical and biochemical mortality predictors, for the most commonly captured species, upon which recreational angling may have the greatest impact.

Based on the literature reviewed here, several key recommendations for best practice stand out. These are likely unsurprising to those involved in recreational angling-based research; however, the empirical data provided here help to underline their importance. In brief, these include the use of corrodible, non-offset, barbless circle-hooks and removal of these where possible, use of appropriately rated fishing gear to minimize fight times, rapid unhooking and release of captured elasmobranchs without removal from the water and avoidance of handling practices or equipment (e.g. gaffs) likely to cause injury to captured elasmobranchs. Such recommendations are only of value if followed by anglers, with angler engagement being key to compliance. Engagement with the angling community may be best improved by provision of angler forums/workshops, led primarily by trusted angling ‘personalities’, in addition to online and physical resources, as this enables guidance to be tailored to individuals and allows anglers’ queries and concerns to be addressed directly. Such an approach would also better facilitate species-specific education and guidance where native species are particularly vulnerable to angling-related impacts, their populations are threatened or in decline, their conservation status is not well known among anglers, and/or they may require specific handling procedures or equipment.

Where a more robust approach is required to meet conservation objectives, regulatory bodies should consider the implementation and enforcement of restrictions or total bans on targeting of vulnerable and poor conservation status species, such as great hammerheads. While comparable regulations governing capture of such species are in place in some areas, such as the US state of Florida, there is strong evidence to suggest these, and similar legislation for other large sharks may be ignored by many anglers fishing illegally (Shiffman *et al.*, 2017) or legally circumvented by keeping sharks in the water (Kilfoil *et al.*, 2017). It may also be necessary to implement temporary fishery closures during periods of extremely high water temperatures, with this likely to be of increasing relevance given current climate projections. Similarly, consideration should be given to implementation of temporary fisheries closures around parturition periods, as is commonplace for many teleosts (e.g. salmonids), particularly where pregnant females, and especially those of threatened and/or declining species, represent a large proportion of elasmobranch catches.

As a final point, the importance of viewing such research in a holistic and angler-inclusive context should not be underestimated. Previous research has highlighted the value of anglers in contributing to conservation and research as both citizen scientists (e.g. Drake *et al.*, 2005; Neat *et al.*, 2015) and stakeholders, with many anglers holding conservation-oriented views (Shiffman *et al.*, 2017). Additionally, anglers often fund conservation and research efforts directly or indirectly (e.g. Barnett *et al.*, 2016; Cooke *et al.*, 2016). Irrespective of mixed views about catch-and-release angling, recreational angling will not cease in the near future, with catch-and-release practices therefore vital to reducing angling-related impacts. For that reason, where possible, scientists should work with—rather than against—anglers to determine and develop evidence-based best practice guidelines and appropriate regulations, enabling realization of their potential value as vectors for conservation (Drake *et al.*, 2005), while minimizing the negative effects outlined here. Likewise, anglers must appreciate that they currently have a major role to play regarding elasmobranch welfare and conservation.

## Data Availability

No new data were generated in support of this research. All data analysed were taken directly from the published literature covered in this review, with the relevant studies cited in Table 2 for reference.

## References

[ref1] Aalbers SA , Wang M, Villafana C, Sepulveda CA (2021) Bigeye thresher shark *Alopias superciliosus* movements and post-release survivorship following capture on linked buoy gear. Fish Res 236: 105857. 10.1016/j.fishres.2020.105857.

[ref2] Adams DH , Borucinska JD, Maillett K, Whitburn K, Sander TE (2015) Mortality due to a retained circle hook in a longfin mako shark *Isurus paucus* (Guitart-Manday). J Fish Dis 38: 621–628. 10.1111/jfd.12277. 24974904

[ref3] Adams KR , Fetterplace LC, Davis AR, Taylor MD, Knott NA (2018) Sharks, rays and abortion: The prevalence of capture-induced parturition in elasmobranchs. Biol Conserv 217: 11–27. 10.1016/j.biocon.2017.10.010.

[ref4] Afonso AS , Hazin FHV, Carvalho F, Pacheco JC, Hazin H, Kerstetter DW, Murie D, Burgess GH (2011) Fishing gear modifications to reduce elasmobranch mortality in pelagic and bottom longline fisheries off Northeast Brazil. Fish Res 108: 336–343. 10.1016/j.fishres.2011.01.007.

[ref5] Anderson BN , Bowlby HD, Natanson LJ, Coelho R, Cortés E, Domingo A, Sulikowski JA (2021) Preliminary estimate of post-release survival of immature porbeagles caught with rod-and-reel in the Northwest Atlantic Ocean. Mar Ecol Prog Ser 660: 153–159. 10.3354/meps13603.

[ref6] Arlinghaus R , Cooke SJ, Lyman J, Policansky D, Schwab A, Suski C, Sutton SG, Thorstad EB (2007) Understanding the complexity of catch-and-release in recreational fishing: an integrative synthesis of global knowledge from historical, ethical, social, and biological perspectives. Rev Fish Sci 15: 75–167. 10.1080/10641260601149432.

[ref7] Awruch CA , Simpfendorfer C, Pankhurst NW (2011) Evaluation and use of a portable field kit for measuring whole-blood lactate in sharks. Mar Freshw Res 62: 694–699. 10.1071/MF10149.

[ref8] Bansemer CS , Bennett MB (2010) Retained fishing gear and associated injuries in the east Australian grey nurse sharks (*Carcharias taurus*): implications for population recovery. Mar Freshw Res 61: 97–103. 10.1071/MF08362.

[ref9] Barnett A , Abrantes KG, Baker R, Diedrich AS, Farr M, Kuilboer A, Mahony T, McLeod I, Moscardo G, Prideaux M et al. (2016) Sportfisheries, conservation and sustainable livelihoods: a multidisciplinary guide to developing best practice. Fish Fish 17: 696–713. 10.1111/faf.12140.

[ref10] Begue M , Clua E, Siu G, Meyer C (2020) Prevalence, persistence and impacts of residual fishing hooks on tiger sharks. Fish Res 224: 105462. 10.1016/j.fishres.2019.105462.

[ref11] Binstock AL , Richards TM, Wells RJD, Drymon JM, Gibson-Banks K, Streich MK, Stunz GW, White CF, Whitney NM, Mohan JA (2023) Variable post-release mortality in common shark species captured in Texas shore-based recreational fisheries. PloS One 18: e0281441. 10.1371/journal.pone.0281441. 36780489 PMC9925081

[ref12] Borucinska J , Kohler N, Natanson L, Skomal G (2002) Pathology associated with retained fishing hooks in blue sharks, *Prionace glauca* (L.), with implications for their conservation. J Fish Dis 25: 515–521. 10.1046/j.1365-2761.2002.00396.x.

[ref13] Borucinska J , Martin J, Skomal G (2001) Peritonitis and pericarditis associated with gastric perforation by a retained fishing hook in a blue shark. J Aquat Anim Health 13: 347–354. 10.1577/1548-8667(2001)013<0347:PAPAWG>2.0.CO;2.

[ref14] Bouyoucos IA , Simpfendorfer CA, Rummer JL (2019) Estimating oxygen uptake rates to understand stress in sharks and rays. Rev Fish Biol Fish 29: 297–311. 10.1007/s11160-019-09553-3.

[ref15] Bouyoucos IA , Talwar BS, Brooks EJ, Brownscombe JW, Cooke SJ, Suski CD, Mandelman JW (2018) Exercise intensity while hooked is associated with physiological status of longline-captured sharks. Conserv. Physiol. 6: coy074. 10.1093/conphys/coy074. 30591841 PMC6301290

[ref16] Bowlby H , Joyce W, Benoit H, Sulikowski J (2020) Evaluation of post-release mortality for porbeagle and shortfin mako sharks from the Canadian pelagic longline fishery. Collect Vol Sci Pap 76: 365–373.

[ref17] Bowlby HD , Benoît HP, Joyce W, Sulikowski J, Coelho R, Domingo A, Cortés E, Hazin F, Macias D, Biais G et al. (2021) Beyond post-release mortality: inferences on recovery periods and natural mortality from electronic tagging data for discarded lamnid sharks. Front Mar Sci 8: 325. 10.3389/fmars.2021.619190.

[ref18] Braccini JM , Waltrick D (2019) Species-specific at-vessel mortality of sharks and rays captured by demersal longlines. Mar Policy 99: 94–98. 10.1016/j.marpol.2018.10.033.

[ref19] Brill R , Bushnell P, Schroff S, Seifert R, Galvin M (2008) Effects of anaerobic exercise accompanying catch-and-release fishing on blood-oxygen affinity of the sandbar shark (*Carcharhinus plumbeus*, Nardo). J Exp Mar Bio Ecol 354: 132–143. 10.1016/j.jembe.2007.10.011.

[ref20] Brownscombe JW , Danylchuk AJ, Chapman JM, Gutowsky LFG, Cooke SJ (2017) Best practices for catch-and-release recreational fisheries—angling tools and tactics. Fish Res 186: 693–705. 10.1016/j.fishres.2016.04.018.

[ref21] Bullock RW , Guttridge TL, Cowx IG, Elliott M, Gruber SH (2015) The behaviour and recovery of juvenile lemon sharks *Negaprion brevirostris* in response to external accelerometer tag attachment. J Fish Biol 87: 1342–1354. 10.1111/jfb.12808. 26511658

[ref22] Campana SE (2016) Transboundary movements, unmonitored fishing mortality, and ineffective international fisheries management pose risks for pelagic sharks in the Northwest Atlantic. Can J Fish Aquat Sci 73: 1599–1607. 10.1139/cjfas-2015-0502.

[ref23] Campana SE , Joyce W, Fowler M, Showell M (2016) Discards, hooking, and post-release mortality of porbeagle (*Lamna nasus*), shortfin mako (*Isurus oxyrinchus*), and blue shark (*Prionace glauca*) in the Canadian pelagic longline fishery. ICES J Mar Sci 73: 520–528. 10.1093/icesjms/fsv234.

[ref24] Campana SE , Joyce W, Manning MJ (2009) Bycatch and discard mortality in commercially caught blue sharks *Prionace glauca* assessed using archival satellite pop-up tags. Mar Ecol Prog Ser 387: 241–253. 10.3354/meps08109.

[ref25] Chin A , Mourier J, Rummer JL (2015) Blacktip reef sharks (*Carcharhinus melanopterus*) show high capacity for wound healing and recovery following injury. Conserv Physiol 3: cov062. 10.1093/conphys/cov062. 27293741 PMC4778477

[ref26] Cicia AM , Schlenker LS, Sulikowski JA, Mandelman JW (2012) Seasonal variations in the physiological stress response to discrete bouts of aerial exposure in the little skate, *Leucoraja erinacea*. Comp Biochem Physiol Part A Mol Integr Physiol 162: 130–138. 10.1016/j.cbpa.2011.06.003. 21689777

[ref27] Clark TD , Sandblom E, Jutfelt F (2013) Aerobic scope measurements of fishes in an era of climate change: respirometry, relevance and recommendations. J Exp Biol 216: 2771–2782. 10.1242/jeb.084251. 23842625

[ref28] Cooke SJ , Donaldson MR, O’connor CM, Raby GD, Arlinghaus R, Danylchuk AJ, Hanson KC, Hinch SG, Clark TD, Patterson DA (2013) The physiological consequences of catch-and-release angling: perspectives on experimental design, interpretation, extrapolation and relevance to stakeholders. Fish Manag Ecol 20: 268–287. 10.1111/j.1365-2400.2012.00867.x.

[ref29] Cooke SJ , Hogan ZS, Butcher PA, Stokesbury MJW, Raghavan R, Gallagher AJ, Hammerschlag N, Danylchuk AJ (2016) Angling for endangered fish: conservation problem or conservation action? Fish Fish 17: 249–265. 10.1111/faf.12076.

[ref30] Cooke SJ , Schramm HL (2007) Catch-and-release science and its application to conservation and management of recreational fisheries. Fish Manag Ecol 14: 73–79. 10.1111/j.1365-2400.2007.00527.x.

[ref31] Cooke SJ , Sneddon LU (2007) Animal welfare perspectives on recreational angling. Appl Anim Behav Sci 104: 176–198. 10.1016/j.applanim.2006.09.002.

[ref32] Cooke SJ , Wilde GR (2007)The fate of fish released by recreational anglers. In SJ Kennelly eds, By-Catch Reduction in the World’s Fisheries. Reviews: Methods and Technologies in Fish Biology and Fisheries, Vol 7. Springer, Dordrecht, pp. 181–234, 10.1007/978-1-4020-6078-6_7.

[ref33] Danylchuk AJ , Suski CD, Mandelman JW, Murchie KJ, Haak CR, Brooks AML, Cooke SJ (2014) Hooking injury, physiological status and short-term mortality of juvenile lemon sharks (*Negaprion bevirostris*) following catch-and-release recreational angling. Conserv. Physiol. 2: cot036. 10.1093/conphys/cot036. 27293620 PMC4732486

[ref34] Dapp DR , Walker TI, Huveneers C, Reina RD (2016) Respiratory mode and gear type are important determinants of elasmobranch immediate and post-release mortality. Fish Fish 17: 507–524. 10.1111/faf.12124.

[ref35] Davie PS , Franklin CE, Grigg GC (1993) Blood pressure and heart rate during tonic immobility in the black tipped reef shark, *Carcharhinus melanoptera*. Fish Physiol Biochem 12: 95–100. 10.1007/BF00004374. 24202688

[ref36] Davis MW (2010) Fish stress and mortality can be predicted using reflex impairment. Fish Fish 11: 1–11. 10.1111/j.1467-2979.2009.00331.x.

[ref37] Diaz GA , Serafy JE (2005) Longline-caught blue shark (*Prionace glauca*): factors affecting the numbers available for live release. Fish Bull 103.

[ref38] Dicken ML , Smale MJ, Booth AJ (2006) Shark fishing effort and catch of the ragged-tooth shark *Carcharias taurus* in the South African competitive shore-angling fishery. African J Mar Sci 28: 589–601. 10.2989/18142320609504209.

[ref39] Domingo A , Casaca Santos C, Carlson J, Natanson L, Cortes E, Mas F, Miller P, Hazin FH, Travassos P, Coelho R (2018) Post-release mortality of shortfin mako in the Atlantic using satellite telemetry: Preliminary results. Report of the 2018 intersessional meeting of the shark species group; SCRS/2018/105.

[ref40] Drake SC , Drake JA, Johnson ML (2005) 2000+ UK Shark Tagging Program: an angler led shark-tagging initiative in UK Coastal Waters. J Northw Atl Fish Sci 35: 233–238. 10.2960/J.v35.m483.

[ref41] Dulvy NK , Pacoureau N, Rigby CL, Pollom RA, Jabado RW, Ebert DA, Finucci B, Pollock CM, Cheok J, Derrick DH et al. (2021) Overfishing drives over one-third of all sharks and rays toward a global extinction crisis. Curr Biol 31: 4773–4787.e8. 10.1016/j.cub.2021.08.062. 34492229

[ref42] Endicott M , Agnew DJ (2004) The survivorship of rays discarded from the South Georgia longline fishery. CCAMLR Sci 11: 155–164.

[ref43] Enever R , Catchpole TL, Ellis JR, Grant A (2009) The survival of skates (Rajidae) caught by demersal trawlers fishing in UK waters. Fish Res 97: 72–76. 10.1016/j.fishres.2009.01.001.

[ref44] Fiedler PC (2010) Comparison of objective descriptions of the thermocline. Limnol Oceanogr Methods 8: 313–325. 10.4319/lom.2010.8.313.

[ref45] Field IC , Meekan MG, Buckworth RC, Bradshaw CJA (2009) Chapter 4 susceptibility of sharks, rays and chimaeras to global extinction. Adv Mar Biol 56: 275–363. 10.1016/S0065-2881(09)56004-X. 19895977

[ref46] Foster DG , Epperly SP, Shah AK, Watson JW (2012) Evaluation of hook and bait type on the catch rates in the western North Atlantic Ocean pelagic longline fishery. Bull Mar Sci 88: 529–545. 10.5343/bms.2011.1081.

[ref47] François P , Sidonie C, Caroline C, Jean-Marc G (2019) The effect of hook type and trailing gear on hook shedding and fate of pelagic stingray (*Pteroplatytrygon violacea*): new insights to develop effective mitigation approaches. Mar Policy 107: 103594. 10.1016/j.marpol.2019.103594.

[ref48] Freire KMF , Belhabib D, Espedido JC, Hood L, Kleisner KM, Lam VWL, Machado ML, Mendonça JT, Meeuwig JJ, Moro PS et al. (2020) Estimating global catches of marine recreational fisheries. Front Mar Sci 7: 12. 10.3389/fmars.2020.00012.

[ref49] French RP (2017) Integrating biological and social information to inform responsible practices for recreational shark fishing. University of Tasmania

[ref50] French RP , Lyle J, Tracey S, Currie S, Semmens JM (2015a) High survivorship after catch-and-release fishing suggests physiological resilience in the endothermic shortfin mako shark (*Isurus oxyrinchus*). Conserv. Physiol. 3: 3. 10.1093/conphys/cov044. PMC4778490 27303650

[ref51] French RP , Lyle JM, Lennox RJ, Cooke SJ, Semmens JM (2019a) Motivation and harvesting behaviour of fishers in a specialized fishery targeting a top predator species at risk. People Nat 1: 44–58. 10.1002/pan3.9.

[ref52] French RP , Lyle JM, Tracey S, Currie S, Semmens JM (2015b) Post-release survival of captured mako sharks: Contributing to developing bestpractice for catch and release game fishing. Institute of Marine and Antarctic Studies, Hobart TAS, p. 7001

[ref53] French RP , Lyle JM, Twardek WM, Cooke SJ, Semmens JM (2019b) A characterization of Australian shortfin mako shark anglers. Mar Policy 110: 103550. 10.1016/j.marpol.2019.103550.

[ref54] Gale MK , Hinch SG, Donaldson MR (2013) The role of temperature in the capture and release of fish. Fish Fish 14: 1–33. 10.1111/j.1467-2979.2011.00441.x.

[ref55] Gallagher AJ , Cooke SJ, Hammerschlag N (2015) Risk perceptions and conservation ethics among recreational anglers targeting threatened sharks in the subtropical Atlantic. Endanger Species Res 29: 81–93. 10.3354/esr00704.

[ref56] Gallagher AJ , Hammerschlag N, Danylchuk AJ, Cooke SJ (2017a) Shark recreational fisheries: Status, challenges, and research needs. Ambio 46: 385–398. 10.1007/S13280-016-0856-8. 27995551 PMC5385669

[ref57] Gallagher AJ , Serafy JE, Cooke SJ, Hammerschlag N (2014) Physiological stress response, reflex impairment, and survival of five sympatric shark species following experimental capture and release. Mar Ecol Prog Ser 496: 207–218. 10.3354/meps10490.

[ref58] Gallagher AJ , Staaterman ER, Cooke SJ, Hammerschlag N (2017b) Behavioural responses to fisheries capture among sharks caught using experimental fishery gear. Can J Fish Aquat Sci 74: 1–7. 10.1139/cjfas-2016-0165.

[ref59] Godin AC , Carlson JK, Burgener V (2012) The effect of circle hooks on shark catchability and at-vessel mortality rates in longlines fisheries. Bull Mar Sci 88: 469–483. 10.5343/bms.2011.1054.

[ref60] Guida L , Awruch C, Walker TI, Reina RD (2017) Prenatal stress from trawl capture affects mothers and neonates: a case study using the southern fiddler ray (*Trygonorrhina dumerilii*). Sci Rep 7: 1–10. 10.1038/srep46300. 28401959 PMC5388872

[ref61] Guida L , Walker TI, Reina RD (2016) The adenylate energy charge as a new and useful indicator of capture stress in chondrichthyans. J Comp Physiol B 186: 193–204. 10.1007/s00360-015-0948-y. 26660290

[ref62] Gulak SJB , Carlson JK (2021) Less Soak Time Saves Those upon the Line: Capture Times and Hooking Mortality of Sharks Caught on Bottom Longlines. North Am J Fish Manag 41: 791–808. 10.1002/nafm.10592.

[ref63] Gurshin CWD , Szedlmayer ST (2004) Short-term survival and movements of Atlantic sharpnose sharks captured by hook-and-line in the north-east Gulf of Mexico. J Fish Biol 65: 973–986. 10.1111/j.0022-1112.2004.00501.x.

[ref64] Hannan KM , Fogg AQ, Driggers WB, Hoffmayer ER, Ingram GW, Grace MA (2013) Size selectivity and catch rates of two small coastal shark species caught on circle and J hooks in the northern Gulf of Mexico. Fish Res 147: 145–149. 10.1016/j.fishres.2013.05.005.

[ref65] Harding L , Gallagher A, Jackson A, Bortoluzzi J, Dolton HR, Shea B, Harman L, Edwards D, Payne N (2022) Capture heats up sharks. Conserv Physiol 10: coac065. 10.1093/conphys/coac065. 36186915 PMC9517936

[ref66] Harter TS , Morrison PR, Mandelman JW, Rummer JL, Farrell AP, Brill RW, Brauner CJ (2015) Validation of the i-STAT system for the analysis of blood gases and acid–base status in juvenile sandbar shark (*Carcharhinus plumbeus*). Conserv. Physiol. 3: cov002. 10.1093/conphys/cov002. 27293687 PMC4778487

[ref67] Heard M , Sutton S, Rogers P, Huveneers C (2016) Actions speak louder than words: tournament angling as an avenue to promote best practice for pelagic shark fishing. Mar Policy 64: 168–173. 10.1016/j.marpol.2015.11.019.

[ref68] Heberer C , Aalbers SA, Bernal D, Kohin S, DiFiore B, Sepulveda CA (2010) Insights into catch-and-release survivorship and stress-induced blood biochemistry of common thresher sharks (*Alopias vulpinus*) captured in the southern California recreational fishery. Fish Res 106: 495–500. 10.1016/j.fishres.2010.09.024.

[ref69] Hight BV , Holts D, Graham JB, Kennedy BP, Taylor V, Sepulveda CA, Bernal D, Ramon D, Rasmussen R, Lai NC (2007) Plasma catecholamine levels as indicators of the post-release survivorship of juvenile pelagic sharks caught on experimental drift longlines in the Southern California Bight. Mar Freshw Res 58: 145–151. 10.1071/MF05260.

[ref70] Hingley L (2020) Conservation Implications of Land-Based Trophy Shark Fishing [Masters thesis]. The Univeristy of Western Australia

[ref131] Holts DB , Bedford DW (1993) Horizontal and vertical movements of the shortfin mako shark, *Isurus oxyrinchus*, in the Southern California Bight. *Marine and Freshwater Research* 44: 901–909. 10.1071/MF9930901.

[ref71] Horst J (2000) Releasing Your Catch. Louisiana State University

[ref72] Hyatt MW , Anderson PA, O’Donnell PM (2016) Behavioral release condition score of bull and bonnethead sharks as a coarse indicator of stress. J Coast Res 32: 1464–1472. 10.2112/JCOASTRES-D-15-00108.1.

[ref73] Iosilevskii G , Kong JD, Meyer CG, Watanabe YY, Papastamatiou YP, Royer MA, Nakamura I, Sato K, Doyle TK, Harman L et al. (2022) A general swimming response in exhausted obligate swimming fish. R Soc Open Sci 9: 211869. 10.1098/rsos.211869. 36147936 PMC9490326

[ref74] Jerome JM , Gallagher AJ, Cooke SJ, Hammerschlag N (2018) Integrating reflexes with physiological measures to evaluate coastal shark stress response to capture. ICES J Mar Sci 75: 796–804. 10.1093/icesjms/fsx191.

[ref75] Keller B , Swimmer Y, Brown C (2020) Review on the Effect of Hook Type on the Catchability, Hooking Location, and Post-Capture Mortality of the Shortfin Mako, Isurus Oxyrinchus

[ref76] Keller BA , Reinhardt JF, Swimmer Y, Brown CA (2021) The Effect of Terminal Gear Modifications on the Total Mortality of the Shortfin Mako, Isurus oxyrinchus

[ref77] Kilfoil JP , Wetherbee BM, Carlson JK, Fox DA (2017) Targeted catch-and-release of prohibited sharks: sand tigers in coastal Delaware waters. Fisheries 42: 281–287. 10.1080/03632415.2017.1306974.

[ref78] Kneebone J , Chisholm J, Bernal D, Skomal G (2013) The physiological effects of capture stress, recovery, and post-release survivorship of juvenile sand tigers (*Carcharias taurus*) caught on rod and reel. Fish Res 147: 103–114. 10.1016/j.fishres.2013.04.009.

[ref79] Knotek RJ , Frazier BS, Daly-Engel TS, White CF, Barry SN, Cave EJ, Whitney NM (2022) Post-release mortality, recovery, and stress physiology of blacknose sharks, *Carcharhinus acronotus*, in the Southeast U.S. recreational shark fishery. Fish Res 254: 106406. 10.1016/J.FISHRES.2022.106406.

[ref80] Lambert FN , Treberg JR, Anderson WG, Brandt C, Evans AN (2018) The physiological stress response of the Atlantic stingray (*Hypanus sabinus*) to aerial exposure. Comp. Biochem Physiol Part A Mol Integr Physiol 219-220: 38–43. 10.1016/j.cbpa.2018.02.009. 29482030

[ref81] Laptikhovsky VV (2004) Survival rates of rays discarded by the bottom trawl squid fishery off the Falkland Islands.

[ref82] Lavender E , Aleynik D, Dodd J, Illian J, James M, Wright PJ, Smout S, Thorburn J (2022) Behavioural responses of a large, benthic elasmobranch to catch-and-release angling. Front Mar Sci 9: 9. 10.3389/fmars.2022.864344.

[ref83] Lécu A , Herbert R, Coulier L, Murray MJ (2011) Removal of an intracoelomic hook via laparotomy in a sandbar shark (*Carcharhinus plumbeus*). J Zoo Wildl Med 42: 256–262. 10.1638/2009-0067.1. 22946403

[ref84] Lynch AJ , Sutton SG, Simpfendorfer CA (2010) Implications of recreational fishing for elasmobranch conservation in the Great Barrier Reef Marine Park. Aquat Conserv Mar Freshw Ecosyst 20: 312–318. 10.1002/aqc.1056.

[ref85] Mandelman JW , Cicia AM, Ingram GW Jr, Driggers WB III, Coutre KM, Sulikowski JA (2013) Short-term post-release mortality of skates (family Rajidae) discarded in a western North Atlantic commercial otter trawl fishery. Fish Res 139: 76–84. 10.1016/j.fishres.2012.09.020.

[ref86] Mandelman JW , Skomal GB (2009) Differential sensitivity to capture stress assessed by blood acid–base status in five carcharhinid sharks. J Comp Physiol B 179: 267–277. 10.1007/s00360-008-0306-4. 18846381

[ref87] Mannheim SL , Childs A-R, Butler EC, Winkler AC, Parkinson MC, Farthing MW, Zweig T, McCord M, Drobniewska N, Potts WM (2018) Working with, not against recreational anglers: evaluating a pro-environmental behavioural strategy for improving catch-and-release behaviour. Fish Res 206: 44–56. 10.1016/j.fishres.2018.04.016.

[ref88] Marshall H , Field L, Afiadata A, Sepulveda C, Skomal G, Bernal D (2012) Hematological indicators of stress in longline-captured sharks. Comp Biochem Physiol Part A Mol Integr Physiol 162: 121–129. 10.1016/j.cbpa.2012.02.008. 22353217

[ref89] McClellan Press K , Mandelman J, Burgess E, Cooke SJ, Nguyen VM, Danylchuk AJ (2016) Catching sharks: recreational saltwater angler behaviours and attitudes regarding shark encounters and conservation. Aquat Conserv Mar Freshw Ecosyst 26: 689–702. 10.1002/aqc.2581.

[ref90] McLoughlin K , Eliason G (2008) Review of information on cryptic mortality and the survival of sharks and rays released by recreational fishers. Canberra, Bur Rural Sci.

[ref91] Mohan JA , Jones ER, Hendon JM, Falterman B, Boswell KM, Hoffmayer ER, Wells RJD (2020) Capture stress and post-release mortality of blacktip sharks in recreational charter fisheries of the Gulf of Mexico. Conserv. Physiol. 8: coaa041. 10.1093/conphys/coaa041. 32440352 PMC7233284

[ref92] Moyes CD , Fragoso N, Musyl MK, Brill RW (2006) Predicting Postrelease Survival in Large Pelagic Fish. Trans Am Fish Soc 135: 1389–1397. 10.1577/T05-224.1.

[ref93] Mucientes G , Queiroz N (2019) Presence of plastic debris and retained fishing hooks in oceanic sharks. Mar Pollut Bull 143: 6–11. 10.1016/j.marpolbul.2019.04.028. 31789167

[ref94] Murray C , Connors R, O’Connor I, Dowling V (2015) The physiological response and recovery of a common elasmobranch bycatch species: The lesser spotted dogfish (*Scyliorhinus canicula*) subject to a controlled exposure event. Biology and Environment: Proceedings of the Royal Irish Academy 143–156.

[ref95] Musyl MK , Gilman EL (2018) Post-release fishing mortality of blue (*Prionace glauca*) and silky shark (*Carcharhinus falciformes*) from a Palauan-based commercial longline fishery. Rev Fish Biol Fish 28: 567–586. 10.1007/s11160-018-9517-2.

[ref96] Musyl MK , Gilman EL (2019) Meta-analysis of post-release fishing mortality in apex predatory pelagic sharks and white marlin. Fish Fish 20: 466–500. 10.1111/faf.12358.

[ref97] Neat F , Pinto C, Burrett I, Cowie L, Travis J, Thorburn J, Gibb F, Wright PJ (2015) Site fidelity, survival and conservation options for the threatened flapper skate (*Dipturus cf. intermedia*). Aquat. Conserv. Mar. Freshw. Ecosyst. 25: 6–20. 10.1002/aqc.2472.

[ref98] NOAA (2003) Fishery management plan for Atlantic tunas, swordfish and sharks. Highly Migratory Species Management Division, Office of Sustainable Fisheries, National Marine Fisheries Service, NOAA, 1315 East-West Highway, Silver Spring, Maryland 20910

[ref99] NOAA (2014) Fisheries of the United States. Fisheries Statistics Division, National Marine Fisheries Service, NOAA, 1315 East-West Highway, Silver Spring, Maryland 20910

[ref100] Otway NM (2020) Capture-induced exertional rhabdomyolysis in the Shortfin Mako Shark, *Isurus oxyrinchus*. Vet Clin Pathol 49: 23–41. 10.1111/vcp.12824. 32090365

[ref101] Pacheco JC , Kerstetter DW, Hazin FH, Hazin H, Segundo R, Graves JE, Carvalho F, Travassos PE (2011) A comparison of circle hook and J hook performance in a western equatorial Atlantic Ocean pelagic longline fishery. Fish Res 107: 39–45. 10.1016/j.fishres.2010.10.003.

[ref102] Prohaska BK , Bethea DM, Poulakis GR, Scharer RM, Knotek R, Carlson JK, Grubbs RD (2018) Physiological stress in the smalltooth sawfish: effects of ontogeny, capture method, and habitat quality. Endanger Species Res 36: 121–135. 10.3354/esr00892.

[ref103] Prohaska BK , Talwar BS, Grubbs RD (2021) Blood biochemical status of deep-sea sharks following longline capture in the Gulf of Mexico. Conserv. Physiol. 9: coaa113. 10.1093/conphys/coaa113. 33505700 PMC7816797

[ref104] Reinhardt JF , Weaver J, Latham PJ, Dell’Apa A, Serafy JE, Browder JA, Christman M, Foster DG, Blankinship DR (2018) Catch rate and at-vessel mortality of circle hooks versus J-hooks in pelagic longline fisheries: a global meta-analysis. Fish Fish 19: 413–430. 10.1111/faf.12260.

[ref105] Renshaw GMC , Kutek AK, Grant GD, Anoopkumar-Dukie S (2012) Forecasting elasmobranch survival following exposure to severe stressors. Comp Biochem. Physiol. Part A Mol. Integr. Physiol. 162: 101–112. 10.1016/j.cbpa.2011.08.001. 21851860

[ref106] Rice PH , Serafy JE, Snodgrass D, Prince ED (2012) Performance of non-offset and 10 offset 18/0 circle hooks in the United States pelagic longline fishery. Bull. Mar. Sci. 88: 571–587. 10.5343/bms.2011.1095.

[ref107] Rodríguez-Cabello C , Sánchez F (2017) Catch and post-release mortalities of deep-water sharks caught by bottom longlines in the Cantabrian Sea (NE Atlantic). J Sea Res 130: 248–255. 10.1016/j.seares.2017.04.004.

[ref108] Scarponi V , Gennari E, Hughes W (2021) Physiological response to capture stress in endemic Southern African catsharks (family Scyliorhinidae). J Fish Biol 99: 186–196. 10.1111/jfb.14710. 33625732

[ref109] Sepulveda CA , Heberer C, Aalbers SA, Spear N, Kinney M, Bernal D, Kohin S (2015) Post-release survivorship studies on common thresher sharks (*Alopias vulpinus*) captured in the southern California recreational fishery. Fish Res 161: 102–108. 10.1016/j.fishres.2014.06.014.

[ref110] Sepulveda CA , Wang M, Aalbers SA (2019) Post-release survivorship and movements of bigeye thresher sharks, *Alopias superciliosus*, following capture on deep-set buoy gear. Fish Res 219: 105312. 10.1016/j.fishres.2019.105312.

[ref111] Serafy JE , Cooke SJ, Diaz GA, Graves JE, Hall M, Shivji M, Swimmer Y (2012) Circle hooks in commercial, recreational, and artisanal fisheries: research status and needs for improved conservation and management. Bull. Mar. Sci. 88: 371–391. 10.5343/bms.2012.1038.

[ref112] Shea BD , Coulter SK, Dooling KE, Isihara HL, Roth JC, Sudal E, Donovan DJ, Hoopes LA, Dove ADM, Cooke SJ et al. (2022) Recreational fishing fight times are not correlated with physiological status of blue sharks (*Prionace glauca*) in the Northwestern Atlantic. Fish Res 248: 106220. 10.1016/j.fishres.2021.106220.

[ref113] Shiffman DS (2020) Recreational shark fishing in Florida: How research and strategic science communication helped to change policy. Conserv Sci Pract 2: e174. 10.1111/csp2.174.

[ref114] Shiffman DS , Macdonald C, Ganz HY, Hammerschlag N (2017) Fishing practices and representations of shark conservation issues among users of a land-based shark angling online forum. Fish Res 196: 13–26. 10.1016/j.fishres.2017.07.031.

[ref115] Skomal GB (2006) The physiological effects of capture stress on post-release survivorship of sharks, tunas, and marlin [Doctoral Dissertation]. Boston University

[ref116] Skomal GB , Chase BC (2002) The physiological effects of angling on post-release survivorship in tunas, sharks, and marlin. American Fisheries Society Symposium 30: 135–138.

[ref117] Skomal GB , Mandelman JW (2012) The physiological response to anthropogenic stressors in marine elasmobranch fishes: a review with a focus on the secondary response. Comp Biochem. Physiol. Part A Mol. Integr. Physiol. 162: 146–155. 10.1016/j.cbpa.2011.10.002. 22008842

[ref118] Spargo AL (2001) The physiological effects of catch and release angling on the post-release survivorship of juvenile sandbar sharks (Carcharhinus plumbeus) [Masters thesis]. University of Rhode Island

[ref119] Weber DN , Frazier BS, Whitney NM, Gelsleichter J, Sancho G (2020) Stress response and post-release mortality of blacktip sharks (*Carcharhinus limbatus*) captured in shore-based and charter boat-based recreational fisheries. Fish Bull 118: 297–314. 10.7755/FB.118.3.8.

[ref120] Weber DN , Janech MG, Burnett LE, Sancho G, Frazier BS (2021) Insights into the origin and magnitude of capture and handling-related stress in a coastal elasmobranch *Carcharhinus limbatus*. ICES J. Mar. Sci. 78: 910–921. 10.1093/icesjms/fsaa223.

[ref121] Weltersbach MS , Strehlow HV (2013) Dead or alive—estimating post-release mortality of Atlantic cod in the recreational fishery. ICES J. Mar. Sci. 70: 864–872. 10.1093/icesjms/fst038.

[ref122] Whitmore BM , White CF, Gleiss AC, Whitney NM (2016) A float-release package for recovering data-loggers from wild sharks. J Exp Mar Bio Ecol 475: 49–53. 10.1016/j.jembe.2015.11.002.

[ref123] Whitney NM , Lear KO, Morris JJ, Hueter RE, Carlson JK, Marshall HM (2021) Connecting post-release mortality to the physiological stress response of large coastal sharks in a commercial longline fishery. PloS One 16: e0255673. 10.1371/journal.pone.0255673. 34525094 PMC8443047

[ref124] Whitney NM , White CF, Anderson PA, Hueter RE, Skomal GB (2017) The physiological stress response, postrelease behavior, and mortality of blacktip sharks (*Carcharhinus limbatus*) caught on circle and J-hooks in the Florida recreational fishery. Fish Bull 115: 532–543. 10.7755/FB.115.4.9.

[ref125] Whitney NM , White CF, Gleiss AC, Schwieterman GD, Anderson P, Hueter RE, Skomal GB (2016) A novel method for determining post-release mortality, behavior, and recovery period using acceleration data loggers. Fish Res 183: 210–221. 10.1016/j.fishres.2016.06.003.

[ref126] Wilde GR , Sawynok W (2009) Effect of hook removal on recapture rates of 27 species of angler-caught fish in Australia. Trans Am Fish Soc 138: 692–697. 10.1577/T08-116.1.

[ref127] Willey AL , Barker LS, Sampson M (2016) A comparison of circle hook and j hook performance in the recreational shark fishery off Maryland. Fish Bull 114: 370–372. 10.7755/FB.114.3.9.

[ref128] Wosnick N , Awruch CA, Adams KR, Gutierre SMM, Bornatowski H, Prado AC, Freire CA (2019) Impacts of fisheries on elasmobranch reproduction: high rates of abortion and subsequent maternal mortality in the shortnose guitarfish. Anim Conserv 22: 198–206. 10.1111/acv.12458.

[ref129] Wosnick N , Navas CA, Niella YV, Monteiro-Filho ELA, Freire CA, Hammerschlag N (2018) Thermal imaging reveals changes in body surface temperatures of blacktip sharks (*Carcharhinus limbatus*) during air exposure. Physiol Biochem Zool 91: 1005–1012. 10.1086/699484. 30074422

[ref130] Yokota K , Kiyota M, Minami H (2006) Shark catch in a pelagic longline fishery: comparison of circle and tuna hooks. Fish Res 81: 337–341.

